# Supplementary material for: A Multianalyte Electrochemical Genosensor for the Detection of High-Risk HPV Genotypes in Oral and Cervical Cancers
Source: Biosensors (Basel). 2022 May 2;12(5):290. doi: 10.3390/bios12050290 (PMC9138520; doi:10.3390/bios12050290)
Supplement: Supplementary file 1 [file biosensors-12-00290-s001.zip › biosensors-1674724-supplementary.pdf]

biosensors

Supporting Information

Article

A Multianalyte Electrochemical Genosensor for the Detection of High-Risk HPV Genotypes in Oral and Cervical Cancers

Thanyarat Chaibun [1,2], Patcharanin Thanasapburachot [3], Patutong Chatchawal [4], Lee Su Yin [5,6], Sirimanas Jiaranuchart [7], Patcharee Jearanaikoon [4], Chamras Promptmas [1], Waranun Buajeeb [2,*] and Benchaporn Lertanantawong [1,*]

1 Biosensors Laboratory, Department of Biomedical Engineering, Faculty of Engineering, Mahidol University, Nakhon Pathom 73170, Thailand; thanyarat.chhh@gmail.com (T.C.); chamras.pro@mahidol.ac.th (C.P.)
2 Department of Oral Medicine and Periodontology, Faculty of Dentistry, Mahidol University, Bangkok 10400, Thailand
3 Research Office, Faculty of Dentistry, Mahidol University, Bangkok 10400, Thailand; patcharanin.tha@mahidol.edu
4 Center for Research and Development of Medical Diagnostic Laboratories, Faculty of Associated Medical Sciences, Khon Kaen University, Khon Kaen 40002, Thailand; patutong@gmail.com (P.C.); patjea@kku.ac.th (P.J.)
5 Faculty of Applied Sciences, AIMST University, Bedong 08100, Malaysia; su_yin@aimst.edu.my
6 Centre of Excellence for Omics-Driven Computational Biodiscovery (ComBio), AIMST University, Bedong 08100, Malaysia
7 Dental Clinic, Chulabhorn Hospital, Chulabhorn Royal Academy and Department of Oral and Maxillofacial Surgery, Faculty of Dentistry, Chulalongkorn University, Bangkok 10330, Thailand, sirimanas.jia@pccms.ac.th
* Correspondence: waranun.bua@mahidol.edu (W.B.); benchaporn.ler@mahidol.ac.th (B.L.)

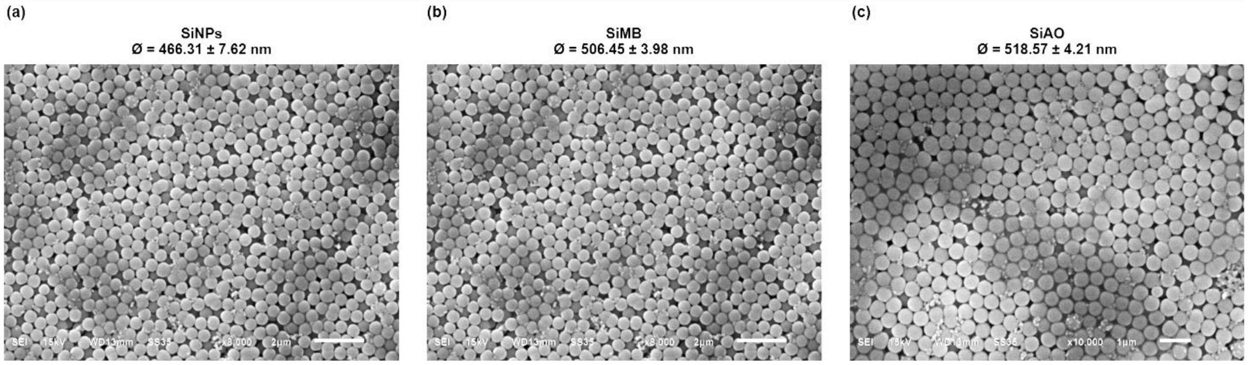

Figure S1: SEM images of the (a) SiNPs (without dye), (b) SiMB and (c) SiAO.
